# Identification and characterization of the first fish parvalbumin-like protein data from a pathogenic fungal species, *Trichophyton violaceum*

**DOI:** 10.1016/j.dib.2020.106420

**Published:** 2020-10-19

**Authors:** Reza Zolfaghari Emameh, Leila Masoori, Hassan Nosrati, Reza Falak, Seppo Parkkila

**Affiliations:** a Department of Energy and Environmental Biotechnology, National Institute of Genetic Engineering and Biotechnology (NIGEB), 14965/161, Tehran, Iran; b Department of Laboratory Sciences, Lorestan University of Medical Sciences, Khorramabad, Iran; c Department of Materials Engineering, Tarbiat Modares University, Tehran, Iran; d Immunology Research Center, Iran University of Medical Sciences, Tehran, Iran; e Faculty of Medicine and Health Technology, Tampere University, FI-33014 Tampere, Finland; f Fimlab Ltd. and Tampere University Hospital, FI-33520 Tampere, Finland

**Keywords:** Polymerase chain reaction (PCR), DNA sequence, Molecular diagnosis, *In silico*, Parvalbumin, Fungi, *Trichophyton violaceum*

## Abstract

Parvalbumins are the most important fish allergens, which are heat-stable, classified in the family of calcium-binding EF-hand proteins, and contain one magnesium binding site. The functional connection between calcium and parvalbumin gives fish the high-speed swimming ability because of high concentration of Ca^2+^-binding parvalbumin in fish white muscles. Although parvalbumins are widely studied and conceivably play crucial roles in the physiology and swimming pattern of fishes, still no report is available about their presence in microbes, such as pathogenic fungal species. We detected a DNA sequence in the genome of *Trichophyton violaceum* and used *in silico* and polymerase chain reaction (PCR) technique with a designed pair of primers to identify it as parvalbumin-coding gene.

## Specifications Table

**Table:** 

Subject area	Biology
Specific subject area	Molecular mycology
Type of data	Figure
How data were acquired	In silico evaluation through databases, molecular detection of the gene sequence by thermocycler (PCR instrument)
Data format	Raw and analyzed
Parameters for data collection	Primers pairs (nucleotide); PCR reaction mixtures ( μl); incubation temperature of T. violaceum (°C); incubation time of T. violaceum (days); PCR product length (bp)
Description of data collection	This molecular detection method was performed in two sections: first was the identification of parvalbumin coding gene sequence of T. violeceum from the database and the second was the designing a primer pair for the coding gene sequence from T. violacum and performing the PCR assay to detect this gene.
Data source location	In silico evaluation of parvalbumin gene sequence from T. violaceum was performed at the National Institute of Genetic Engineering (NIGEB, Tehran, Iran) and the culture of T. violaceum and PCR-based detection were performed at the Iran University of Medical Sciences (Tehran, Iran)
Data accessibility	The UniProt ID of parvalbumin from T. violaceum is A0A178F7E4 (https://www.uniprot.org/uniprot/A0A178F7E4)
Related research article	R. Zolfaghari Emameh, L. Masoori, RA.Taheri, R. Falak, Identification and characterization of parvalbumin-like protein in Trichophyton violaceum, Fungal Biol. 124 (2020) 592-600 https://doi.org/10.1016/j.funbio.2020.02.014

## Value of the Data

• The detected parvalbumin-coding gene and encoded protein may play a crucial role in the physiology and biological functions in *T. violaceum*.
• In addition to direct microscopic detection of *T. violaceum*, parvalbumin-coding gene can be a targeted in the molecular diagnosis of *T. violaceum* by medical mycology laboratories.
• Parvalbumin-coding gene can be a molecular marker for differentiation between *T. violaceum* and other *Trichophyton* species.
• Detection of parvalbumin-coding gene from *T. violaceum* by the PCR assay can decrease the diagnosis time of this pathogenic fungus and play an important role in the health quality of the society.

## 1 Data Description

The multiple sequence alignment (MSA) between the sequence of PCR product (VIOLA_3_F_VIOLA3) and the coding gene for parvalbumin (UniProt ID: A0A178F7E4) from *T. violaceum* showed a high similarity (Raw data of both sequences and MSA analysis were shown in Supplementary file 1). Therefore, the coding gene for parvalbumin of *T. violaceum* was retrieved from UniProt database [1] with the ID number A0A178F7E4, which can be detected in the biological samples by specific primers and PCR as a molecular-based detection technique. To perform a PCR assay, a primer pair was designed using NCBI Primer‐BLAST tool (https://www.ncbi.nlm.nih.gov/tools/primer-blast/) [2] for this coding gene as following: the forward primer is 5′‐ATGGCCTTCAGCAGTGTTCT‐3′ and the reverse primer is 3′‐TTATTGCTTTACAAGGGCAGCA‐5′, and the predicted PCR product length is 330 bp (Raw data of the PCR assay was shown in Supplementary file 2).

## 2 Experimental Design, Materials and Methods

### 2.1 Fungal culture and DNA extraction

*T. violaceum* was cultured on Sabouraud's dextrose agar (SDA, Merck, Germany) at 25 °C for 21–28 days until the Lactophenol Cotton Blue mount colonies were formed. Consequently, the Wizard® Genomic DNA Purification Kit (Promega, Madison, WI, USA) was employed to extract DNA from the defined fungal colonies.

### 2.2 PCR assay and DNA sequencing

**Fig. 1:**
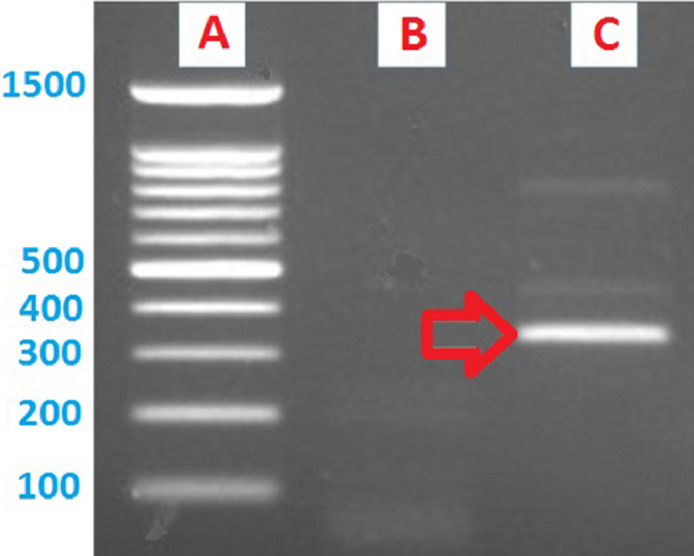
The amplified PCR products for the parvalbumin gene of *T. violaceum*. (A) 100 bp DNA ladder; (B) negative control contains no extracted DNA; and (C) The extracted DNA from *T. violaceum* amplified with the primer pair 2, product size: 330 bp.

To perform the PCR assay for parvalbumin coding gene (UniProt ID: A0A178F7E4) from *T. violaceum*, the following reaction mixture was employed: 12.5  μl of 2X KAPA ReadyMix (KAPA 2G Robust HotStart ReadyMix PCR Kit, Kapa Biosystems, Wilmington, MA, USA), 1.25  μl of each forward and reverse primers (Bioneer Inc, South Korea), 9  μl of dH_2_O and 1  μl of extracted DNA. Then, the PCR product was run on the agarose gel electrophoresis (Fig. 1) and consequently the PCR product or corresponding band for the parvalbumin gene was cut and sequenced by a previously described protocol [3].

### 2.3 In silico *analysis*

**Fig. 2:**
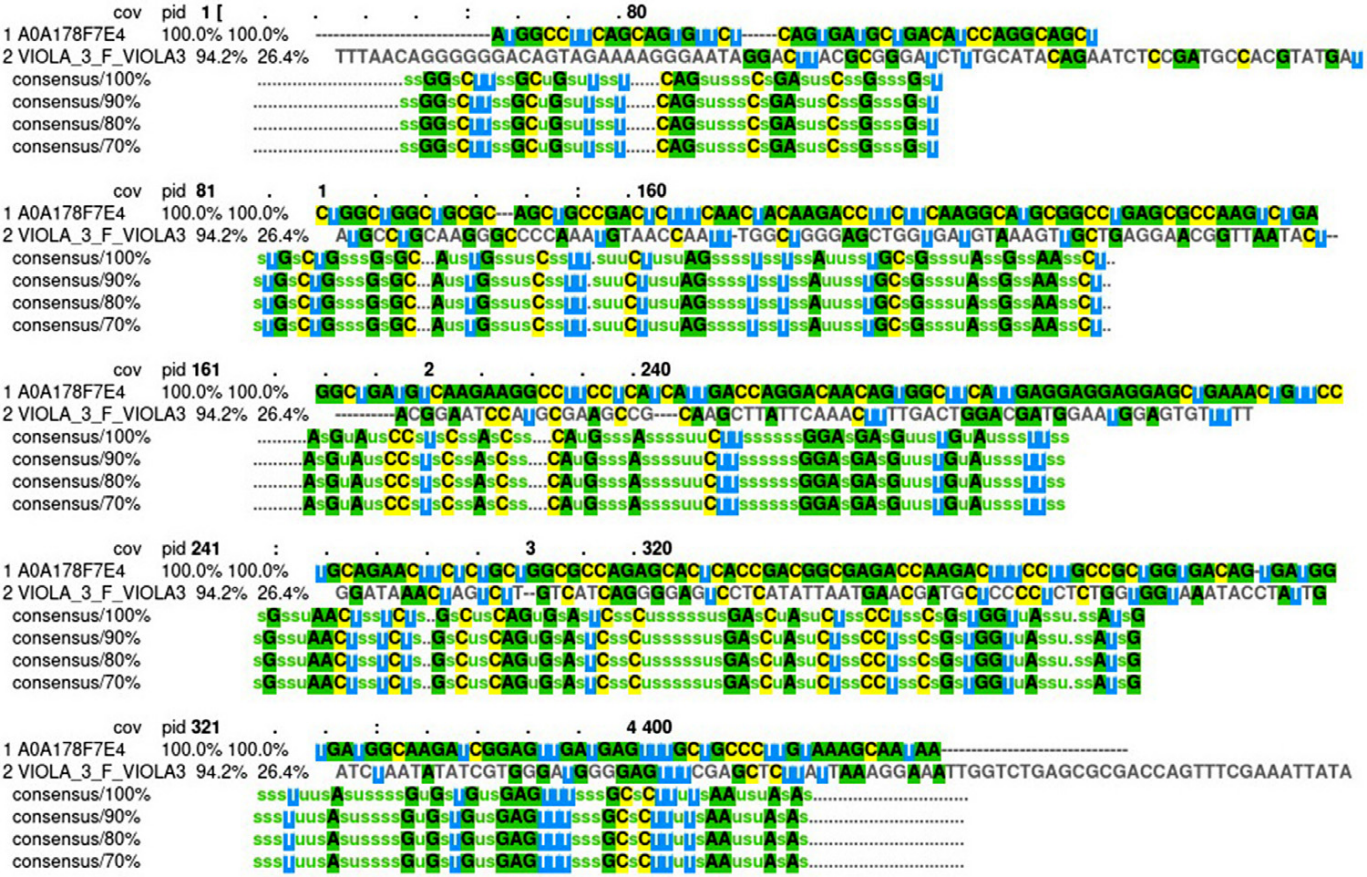
The multiple sequence alignment (MSA) analysis between the sequence of the PCR product (VIOLA_3_F_VIOLA3) and the coding gene for parvalbumin (A0A178F7E4) from *T. violaceum.*

The MSA analysis was performed between the sequenced PCR product (VIOLA_3_F_VIOLA3) and the reference parvalbumin coding gene (UniProt ID: A0A178F7E4) using MView 1.63 program (https://www.ebi.ac.uk/Tools/msa/mview/) (Fig. 2) [4].

## Declaration of Competing Interest

The authors declare that they have no known competing financial interests or personal relation-ships that could have appeared to influence the work reported in this paper.

## Appendix Supplementary materials

Image, application 1

Image, application 2

## Footnotes

Supplementary material associated with this article can be found in the online version at doi:10.1016/j.dib.2020.106420.
