# Supplementary material for: Identification and characterization of the first fish parvalbumin-like protein data from a pathogenic fungal species, Trichophyton violaceum
Source: Data Brief. 2020 Oct 19;33:106420. doi: 10.1016/j.dib.2020.106420 (PMC7586069; doi:10.1016/j.dib.2020.106420)
Supplement: Supplementary file 1 [file mmc1.docx]

**Supplementary file 1:**

> A0A178F7E4 Trichophyton violaceum putative parvalbumin beta

ATGGCCTTCAGCAGTGTTCTCAGTGATGCTGACATCCAGGCAGCTCTGGCTGGCTGCGCA

GCTGCCGACTCTTTCAACTACAAGACCTTCTTCAAGGCATGCGGCCTGAGCGCCAAGTCT

GAGGCTGATGTCAAGAAGGCCTTCCTCATCATTGACCAGGACAACAGTGGCTTCATTGAG

GAGGAGGAGCTGAAACTGTTCCTGCAGAACTTCTCTGCTGGCGCCAGAGCACTCACCGAC

GGCGAGACCAAGACTTTCCTTGCCGCTGGTGACAGTGATGGTGATGGCAAGATCGGAGTT

GATGAGTTTGCTGCCCTTGTAAAGCAATAA

1. **Raw data:** FASTA CDS sequence for *T. violaceum* parvalbumin

>VIOLA_3_F_VIOLA3 sequence exported from VIOLA_3_F_VIOLA3.ab1

TTTAACAGGGGGGACAGTAGAAAAGGGAATAGGACTTACGCGGGATCTTTGCATACAGAA

TCTCCGATGCCACGTATGATATGCCTGCAAGGGCCCCAAATGTAACCAATTTGGCTGGGA

GCTGGTGATGTAAAGTTGCTGAGGAACGGTTAATACTACGGAATCCATGCGAAGCCGCAA

GCTTATTCAAACTTTTGACTGGACGATGGAATGGAGTGTTTTTGGATAAACTAGTCTTGT

CATCAGGGGAGTCCTCATATTAATGAACGATGCTCCCCTCTCTGGTGGTAAATACCTATT

GATCTAATATATCGTGGGATGGGGAGTTTCGAGCTCTTATTAAAGGAAATTGGTCTGAGC

GCGACCAGTTTCGAAATTATAATTTATAACTTATTACCACCACCTTAAATATGAACAGAG

AATTTATCTTTGTGAGGTTTTATTTACCGTTGAGTAGATTCTTTTCTAAGTCCCTCCCGG

GGGTAGCCGGAAGTAGATAA

1. **Raw data:** The sequenced PCR product (VIOLA_3_F_VIOLA3)


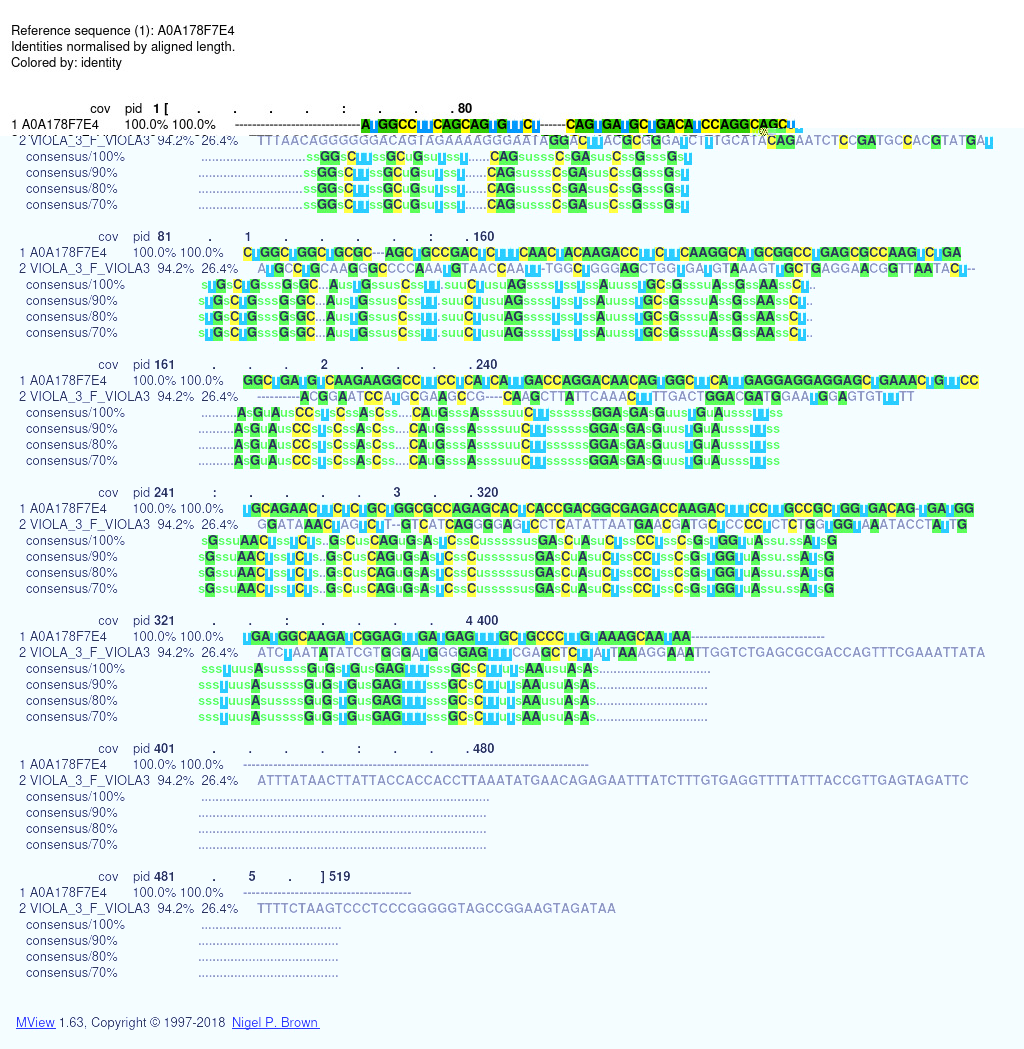


**(C) Raw data:** The MSA analysis between the sequence of the PCR product (VIOLA_3_F_VIOLA3) and the coding gene for parvalbumin (A0A178F7E4) from *T. violaceum.*
