# Supplementary material for: Identification and characterization of the first fish parvalbumin-like protein data from a pathogenic fungal species, Trichophyton violaceum
Source: Data Brief. 2020 Oct 19;33:106420. doi: 10.1016/j.dib.2020.106420 (PMC7586069; doi:10.1016/j.dib.2020.106420)
Supplement: Supplementary file 2 [file mmc2.docx]

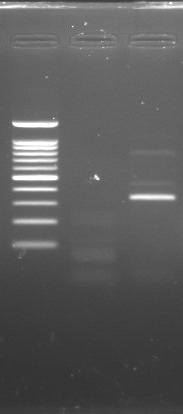


**Supplementary file 2.**

**Raw data:** The amplified PCR products for the parvalbumin gene of *T. violaceum.*
